# Pollution in the Arctic Ocean: An overview of multiple pressures and implications for ecosystem services

**DOI:** 10.1007/s13280-021-01657-0

**Published:** 2021-12-07

**Authors:** Bryony L. Townhill, Efstathios Reppas-Chrysovitsinos, Roxana Sühring, Crispin J. Halsall, Elena Mengo, Tina Sanders, Kirsten Dähnke, Odile Crabeck, Jan Kaiser, Silvana N. R. Birchenough

**Affiliations:** 1 The Centre for Environment, Fisheries and Aquaculture Science (Cefas), Pakefield Road, Lowestoft, Suffolk, NR33 0HT UK; 2 Lancaster Environment Centre, Lancaster University, Lancaster, LA1 4YQ UK; 3 Department of Environmental Science, Stockholm University, 106 91, Stockholm, Sweden; 4 Department of Chemistry and Biology, Ryerson University, Toronto, ON M5B 2K3 Canada; 5 Helmholtz-Zentrum Hereon, Institute for Carbon Cycles, Max-Planck-Str. 1, 21502 Geesthacht, Germany; 6 Centre for Ocean and Atmospheric Sciences, School of Environmental Sciences, University of East Anglia, Norwich, NR4 7TJ UK

**Keywords:** Chemicals, Contaminants, Ecopath, Management, Modelling, Policy

## Abstract

The Arctic is undergoing unprecedented change. Observations and models demonstrate significant perturbations to the physical and biological systems. Arctic species and ecosystems, particularly in the marine environment, are subject to a wide range of pressures from human activities, including exposure to a complex mixture of pollutants, climate change and fishing activity. These pressures affect the ecosystem services that the Arctic provides. Current international policies are attempting to support sustainable exploitation of Arctic resources with a view to balancing human wellbeing and environmental protection. However, assessments of the potential combined impacts of human activities are limited by data, particularly related to pollutants, a limited understanding of physical and biological processes, and single policies that are limited to ecosystem-level actions. This manuscript considers how, when combined, a suite of existing tools can be used to assess the impacts of pollutants in combination with other anthropogenic pressures on Arctic ecosystems, and on the services that these ecosystems provide. Recommendations are made for the advancement of targeted Arctic research to inform environmental practices and regulatory decisions.

## Introduction

The Arctic region is exposed to a range of human pressures of local, regional and global origin, that demonstrate significant perturbations to the Arctic marine ecosystems (Box et al., 2019; Overland et al., 2019). These include pollution from a range of sources, fishing and climate change (Wassman et al., 2011; Macdonald et al., 2017; Huntington et al., 2020). These pressures are a cause for concern both regionally, for the indigenous and local communities that rely on the resources provided by the marine ecosystems, as well as internationally due to the high biological, cultural and economic significance of the Arctic region (CAFF, 2015; Huntington et al., 2015). Over past decades, the human uses of Arctic ecosystems have intensified due to multiple factors such as increased accessibility, particularly in regions that were previously ice-covered for large parts of the year (Anisimov et al., 2007). This expansion of human activity northwards alters highly sensitive Arctic ecosystems and, consequently, compromises the delivery of the ecosystem services they provide (Afflerbach et al., 2017; Huntington et al., 2020).

Sources of aquatic pollution in the Arctic include wastewater and waste from settlements, riverine nutrient inputs caused by thawing permafrost and erosion (Tank et al., 2012), emissions from increasing tourism and shipping, long-range atmospheric and oceanic pollution, commercial fisheries, and chemical and waste emissions from resource exploitation including mining, minerals, oil and gas extraction (AMAP, 2018).

Fishing is an important activity for the region, and in the Barents Sea alone, around 15 million tonnes of fish are caught each year (ICES, 2019). There is a smaller whaling industry (ICES, 2019) as well as aboriginal subsistence whaling (IWC, 2020). Currently, industrial fisheries in some areas of the Arctic are dominated by Inuit communities (Tai et al., 2019). However, catch potential is projected to increase in the Arctic (Cheung et al., 2010) and it is not clear whether this will benefit local communities or international fishing fleets.

The pressures from increased anthropogenic activity are exacerbated by climate change that has affected the Arctic much more severely than many temperate regions. For example, averaged Arctic near-surface air temperatures have increased by 3.1 °C in the last 40 years; three times faster than the global average (AMAP, 2021). Major effects of climate change on the Arctic Ocean are decreasing extent and thickness of sea ice, increasing sea surface temperatures and cloud cover, increased precipitation, increased freshwater influx, decreasing pH and rising sea levels (Meredith et al., 2019; AMAP, 2021). These changes are reported or expected to lead to ecological impacts such as increased primary productivity, decreased calcification rates of some shell-forming organisms, spread of invasive species, spread of pathogens/diseases changes in fish distributions, change in community composition and food web structure, and impacts to marine mammal communities (Macdonald et al., 2005; Wassmann, 2011; Rogers and Laffoley, 2013; Meredith et al., 2019; VanWormer et al., 2019; Huntington et al., 2020; AMAP, 2021). Melting ice is also opening up new Arctic shipping routes (Melia et al., 2017), with associated risks from oil spills. These changes could lead to repercussions for biodiversity, fisheries and local foods and livelihoods for indigenous Arctic communities (Søreide et al. 2010; Johansen et al., 2013; Meredith et al., 2019; AMAP, 2021). There is also evidence that microplastics are released into the ocean when ice melts (Halsband and Herzke, 2019). In Svalbard this coincided with the ice-edge bloom, meaning that microplastics and associated chemicals could be highly bioavailable and enter the food chain (von Friesen et al., 2020).

Managing multiple pressures in the Arctic is particularly challenging due to the multi-national and geopolitical interests, complex legislation and regulation of the region (Platjouw, 2019). Sound scientific research and science–stakeholder interaction is needed to safeguard the Arctic environment and health of local communities while allowing for industrial activities.

To address pan-Arctic regulatory and management questions, the Arctic Council was established in 1996 (Arctic Council, 2021). The Arctic Council is an intergovernmental forum established to promote cooperation, coordination and interaction among the Arctic States, Arctic Indigenous peoples and other Arctic inhabitants on pan-Arctic issues, particularly on issues related to sustainable development and environmental protection in the Arctic (Arctic Council, 2021). However, the Arctic Council is an advisory panel and does not have the power to mandate the implementation of its guidelines and recommendations. This responsibility lies with the independent Arctic States (Arctic Council, 2021). In its advisory role, the Arctic Council has established working groups to identify pollution pressures on Arctic ecosystems and communities (Arctic Council, 2021). Yet, many questions around pollution and multiple pressures are still to be answered by the scientific community and to be reflected in regulatory decision making and management of the Arctic. Some of the pressing questions that need to be addressed include the following:

- How to identify new pressures on the Arctic ecosystem, including emerging pollutants from Arctic and non-Arctic sources?
- How to determine what effects the combination of pressures is having at an ecosystem level?
- How these multiple pressures affect the ecosystem services provided by the Arctic, and ultimately impact society?

In this paper, we aim to describe how these questions can be answered for the Arctic, here defined as the area north of the Arctic Circle, by (1) discussing the utility of different tools for the assessment of ecosystem impacts of pollutants on the Arctic Ocean as part of a system with multiple pressures; (2) identifying ecosystem services that may be affected by pollutant exposure; and (3) identifying methodological and data needs to inform regulatory decisions.

## Tools to assess risks and impacts

Accurately representing the potential synergistic or antagonistic interactions of multiple pollution pressures on ecosystems and human health has increasingly become a focus for research and regulatory risk assessments. In a 2018 “horizon scanning” exercise among members of the Society of Environmental Toxicology and Chemistry (Setac; Villa et al., 2017; Van den Brink et al., 2018), accurate evaluation and representation of multiple pressures were identified among the top three research questions for ensuring sustainable environmental quality in Europe (Van den Brink et al., 2018).

**Fig. 1:**
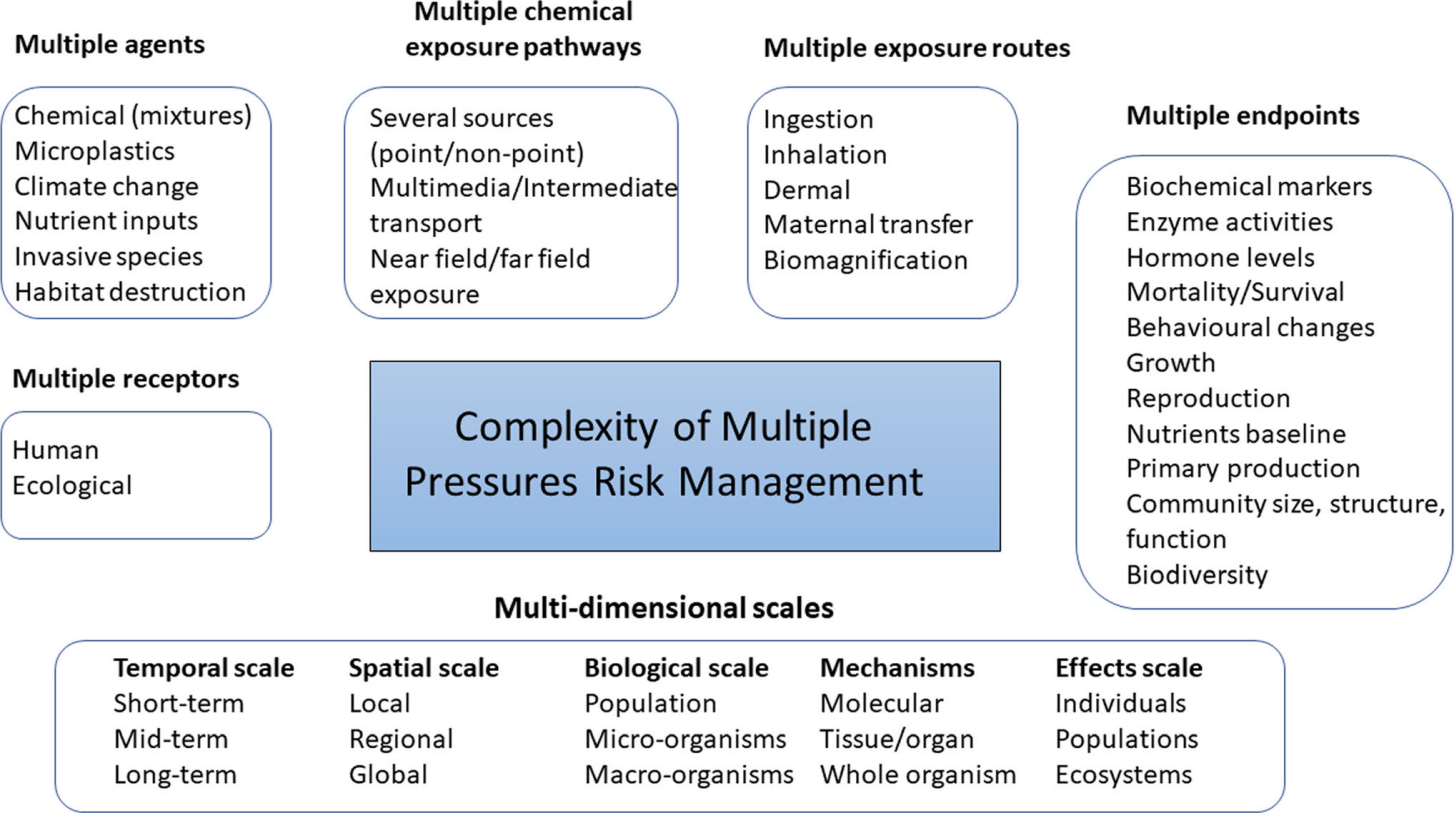
Environmental pollution is a multidimensional risk management challenge that requires integration of information on processes and impacts at several levels. Developed from ideas by van Leeuwen (2007)

Analysing the risks that environmental pollutants pose is complex and multidimensional (Fig. 1). There are many different agents, receptors, routes to exposure and endpoints, as well as scales which need to be considered. To facilitate a multiple pressure analysis for measuring potential risks and realised impacts in the Arctic, there is a need for observational and experimental data, as well as in silico data analysis methods and predictive models. These can then be used to identify risks and quantify impacts on ecosystem services.

### Role and use of observational and experimental data

Observational and experimental data of the ecosystem health, pollutant loads, biogeochemical status, climate, and changes thereof are crucial for our understanding and the sustainable management of Arctic ecosystems. For observational data, the Arctic Council has scientific working groups that collate relevant monitoring data (AMAP, 2018). While these assessment programmes do not collect additional primary scientific data, their reports are invaluable meta-analyses of state-of-the-art Arctic research. Arctic contamination issues are assessed by the Arctic Monitoring and Assessment Program, AMAP (2020) which provides expert scientific evaluations and policy advice on pollutant issues ranging from ocean acidification to organic contaminants of emerging Arctic concern. There are also working groups aimed at reducing Arctic pollution (Arctic Contaminants Action Program, ACAP) and ensuring sustainable development. Individually, the working groups provide information on pressures and effects for a variety of pollutants, biogeochemical cycles, and flora and fauna populations (ACAP, 2020). AMAP is currently completing an assessment of Arctic pollutants and climate change including climate–contaminant interactions and climate–ecosystem interactions (AMAP, 2021). However, the impacts of contaminants and nutrients as multiple pollution pressures on Arctic ecosystems are not part of any working group’s mandate and there are few experimental and observational datasets that include nutrients and contaminants as multiple pollution pressures.

One example trying to bridge this observational gap are experimental facilities such as sea ice chambers. Such facilities allow for the mechanistic evaluation of processes that drive transport and accumulation of different contaminants in sea ice using controlled laboratory or mesocosm type conditions. There are a number of facilities globally, including, for example, the Ocean Sea Ice Mesocosm (OSIM) Facility as part of the Churchill Marine Observatory located at Hudson Bay in Manitoba, Canada (https://umanitoba.ca/environment-earth-resources/earth-observation-science/marine-observatory) (e.g. sea ice and interactions with microplastics and oil: Firoozy et al., 2018; Geilfus et al., 2019), and the Arctic Environmental Test Basin of the Hamburg Ship Model Basin (HSVA) in Hamburg, Germany (https://www.hsva.de/our-facilities/ice-tank.html). An example of a smaller laboratory-based facility aiming to simulate the full system of atmosphere, sea ice and sea water is the Roland von Glasow Air-Sea-Ice Chamber at the University of East Anglia, UK (https://www.uea.ac.uk/about/school-of-environmental-sciences/research/atmosphere-ocean-and-climate-sciences/roland-von-glasow-air-sea-ice-chamber) (Thomas et al., 2021), which has been used recently for studies on organic contaminant fate in sea ice. Studies include work on contaminant accumulation in ice brine by Garnett et al. (2019, 2021a) and the investigation of transport mechanisms of tracers in combination with nutrient inputs by Thomas et al. (2020) as part of the Effects of Ice Stressors and Pollutants on the Arctic marine Cryosphere (EISPAC project), under the UK/German “Changing Arctic Ocean” Programme (https://www.changing-arctic-ocean.ac.uk/ NERC, 2021). These studies help underpin observations in the field with regards to contaminant accumulation in ice and subsequent release during periods of ice thawing (Garnett et al., 2021b).

For coastal environments, the Helmholtz-Zentrum Hereon is currently developing the “Coastal Pollution Toolbox”. This toolbox uses a combination of observational and in silico pollution, nutrient, and oceanographic data to enable the analysis of contaminant and nutrient transport pathways and interactions in temperate and polar coastal zones (https://hzg.de/ms/coastalpollutiontoolbox/index.php.en).

In addition to these experimental datasets, the European Union Earth Observation Programme Copernicus provides a number of open-access satellite remote sensing data on large-scale environmental processes in the Arctic (www.copernicus.eu) (Table 1). These large-scale data sets are needed to provide data on climatic change, high-quality data for model validation, as well as pan-Arctic context for local or regional multiple pressures assessments.

**Table 1:** Examples of data provided by Copernicus which can be useful for studies on multiple pressure and climate change assessments

Copernicus Service	Data available
Copernicus Marine Environment Monitoring Service (CMEMS, 2016)	Observational and forecasting data on currents, temperature, wind, salinity, sea level, sea ice and biogeochemistry
The Copernicus Atmosphere Monitoring Service (CAMS Catalogue, 2020)	Observational and modelling data on atmospheric processes and solar radiation
Copernicus Climate Change service (C3S, 2020)	Monthly sea ice maps for both the Arctic and Antarctic seas
Copernicus Land Monitoring Service (CLMS; Copernicus Service Information, 2021)	High-resolution data on land cover and freshwater

### Added value of in silico data analysis methods and predictive models

**Fig. 2:**
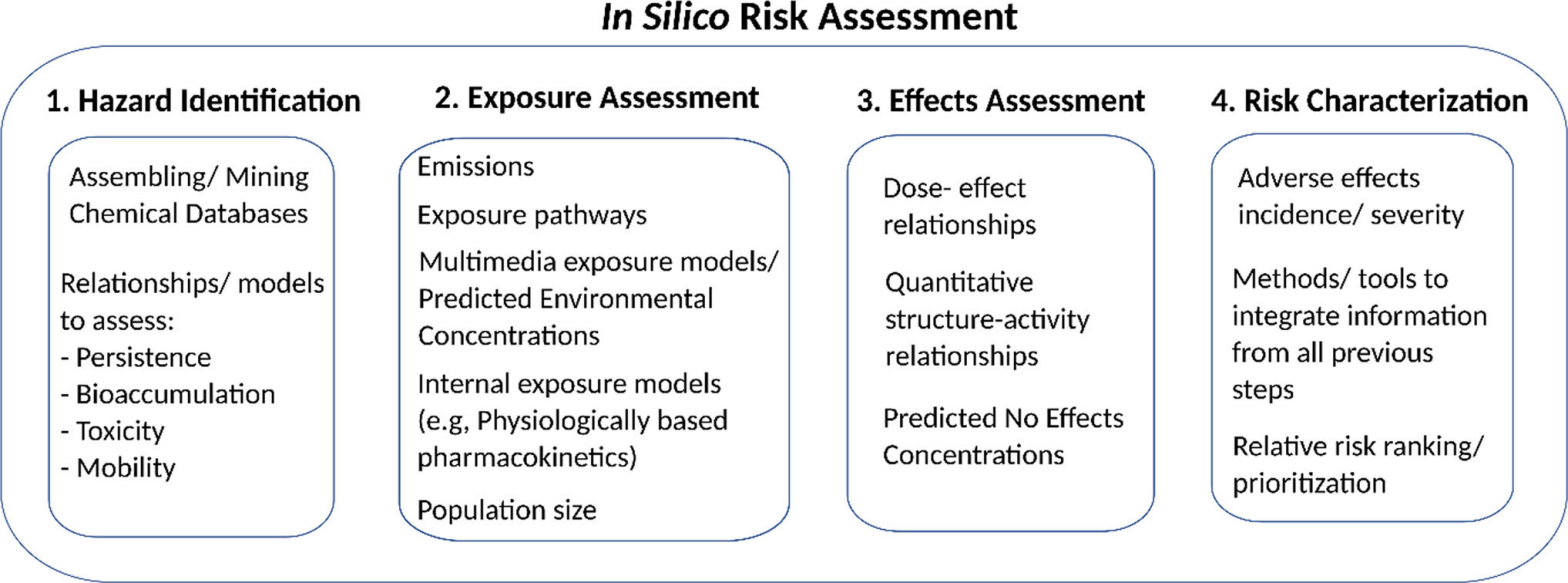
In silico tools and methodologies to perform a chemical risk assessment, for each of the risk assessment steps. Adapted from van Leeuwen (2007) and Reppas Chrysovitsinos (2017)

Performing a chemical risk assessment for the changing Arctic Ocean while accounting for multiple stressors is challenging and practically impossible without in silico tools and techniques. In silico tools facilitate the risk characterisation of multiple pressures by (i) enabling the integration of empirical data and current knowledge on processes and impacts at multiple levels (Fig. 2) (van Leeuwen, 2007) to assess the hazards, exposure, effects for the combined pressures on the Arctic ecosystem, and (ii) unravelling the many effects of multiple pressures and their interactions. Nevertheless, multi-pressure contaminant risk assessment remains a challenge even for sophisticated in silico tools and, thus, modelling efforts frequently focus on performing relative risk or impact assessments and prioritisation exercises for individual pollutants or pressures rather than assessing the risk of multiple pressures. The lack of models that are specifically designed to inform regulatory decision making and management of pollutant impacts in a multiple pressure context means that a combination of models must be used to address different aspects of questions, and/or existing models must be adapted to allow for multiple stressor analyses.

One model that allows multiple stressors to be mapped to perform cumulative human impact assessments and determine the relative importance of key stressors was developed by Halpern et al. (2008). This method spatially maps each pressure and calculates the potential cumulative impact on ecosystem components, determining the regions with the highest impact (Anderson et al., 2020). There are also ecosystem models that explore the impact of single or multiple environmental pressures and the nature of their interactions, with a targeted understanding of key species’ responses and the overarching food-web structure. However, these models are usually not designed to predict the environmental fate and transport of contaminants (Table 2). There are also models specifically designed to evaluate environmental fate, transport and potential risk of organic pollutants from a regulatory perspective (Table 2). Such fate and transport models are useful tools for the screening of environmental transport and risks of organic contaminants such as persistent organic pollutants, however, they are generally not designed to analyse the contaminant impact on local ecosystems.

**Table 2:** The models available that can be used to consider pollutants and multiple pressures from a policy or regulatory perspective

Model	Description	Uses
Ecosystem models		
Ecopath	EwE is an ecosystem model based on (bio)mass-balance calculations and can be used to model scenarios of different pressures (Polovina, 1984; Christensen and Pauly, 1992; Christensen and Walters, 2004; Fulton, 2010)	Integrated ecosystem assessments and multispecies fisheries management (e.g. ICES, 2018a). Test marine protected area management, develop ecological indicators and investigate climate change impacts (e.g. Shannon et al., 2004; Bentley et al., 2017; Serpetti et al., 2017; ICES, 2018b). Multiple pressure assessment (e.g. Corrales et al., 2017; Chagaris et al., 2020)
Atlantis	Atlantis is an end-to-end ecosystem model. Models for Iceland and the Nordic and Barents Seas can be forced with physical variables and provide spatial outputs of biomass, age, numbers, predation, mortality and catches (Hansen et al., 2016; 2019a, 2019b; ICES, 2017; Sturludottir et al., 2018)	Investigate large-scale management options (Fulton, 2010). Test the impact of pollution, such as the oil spill in the Gulf of Mexico (e.g. Ainsworth et al., 2018)
Fate and transport models		
European Union System for the Evaluation of Substances (EUSES)	A multimedia mass-balance fate and transport model	Screen regulatory assessment endpoints such as predicted environmental concentration (PEC) to predict no effect concentration (PNEC), the expected receiving environments and species at risk, the expected persistence, long-range transport potential and efficiency of transport
Risk Assessment IDentification And Ranking (RAIDAR)	Fate and transport model. A multimedia fugacity mass-balance model (Arnot and Mackay, 2008)	Screen regulatory assessment endpoints such as PEC and PNEC, and the expected receiving environments and species at risk
OECD persistence and long-range transport screening tool	Fate and transport model (Wegmann et al., 2009)	Screen regulatory assessment endpoints such as the expected persistence, long-range transport potential and efficiency of transport
BETR-Global	BETR-Global is a geographically explicit global-scale multimedia fugacity mass-balance fate and transport model. (Macleod et al., 2011)	Has been used to study interactions between climate change and exposure to persistent pollutants in Europe and the Arctic (e.g. Wöhrnschimmel et al., 2013)

Bioaccumulation models are used to calculate chemical concentrations in different trophic levels, individual species and ultimately human exposure. Exposure concentrations (or doses) can be compared to toxicity threshold concentrations [e.g. predict no effect concentrations (PNECs)] and the subsequent risk from a specific chemical or chemicals can be predicted (ECHA, 2018). However, these models focus exclusively on the direct chemical impacts and do not allow for the evaluation of non-chemical multiple pressure impacts, such as climate change which in turn may affect chemical exposure and effect.

To the best of our knowledge, there is currently only one available model that allows for the combined analysis of chemical, physical and ecological pressures and their implications for marine ecosystems and their sustainable management: Ecopath with Ecosim (EwE). EwE models enable the evaluation of impacts of different management strategies on the food web, and estimate how a combination of pressures affect key species (ICES, 2018a). Some of these pressures assessed include fishing, climate change, nutrients, noise, shipping, pollutants, physical modifications and non-native species (Corrales et al., 2017; Andersen et al., 2020; Chagaris et al., 2020). Within the EwE suite is “Ecotracer”; a tool that traces the transfer of pollutants through the food web, based on the trophic interactions described in EwE (Walters and Christensen, 2018). Ecotracer can handle a wide range of environmental contaminants, such as organic chemicals, metals, microplastics and radionuclides, and due to the detailed description and dynamic simulation of the food web provided within EwE, it enables an ecosystem-level assessment of bioaccumulation and biomagnification (Walters and Christensen, 2018).

In addition, to bridge the gap between contaminant exposure and biogeochemistry, a tool has recently been developed which links Ecopath with a physical/biogeochemical model (Beecham et al., 2015), enabling lower trophic levels to be simulated by climate scenarios which then feed into Ecopath. This allows simulated temperature changes to propagate through the food web from plankton to the higher trophic levels. This is particularly relevant in the context of the Arctic Ocean and coastal seas, due to the marked seasonality in primary productivity brought about by seasonal changes in sunlight and sea ice cover and the effects of climate change on this productivity (Wassmann, 2011; Wassmann and Reigstad, 2011). In combination, the EwE models thus have the potential to be the ideal tools for analysing the interplay of nutrient and chemical pollution from a regulatory and management perspective.

## Linking ecosystem changes to human wellbeing

While a suite of models and data have been developed to unravel the effects of a wide range of pressures on the Arctic, understanding how these pressures impact on the delivery of Arctic ecosystems services that benefit human wellbeing remains a challenge (Neumann et al., 2019). These effects are subject to complex human–nature dynamics and relations at global, regional and local scale and have impacts over multiple and diverse values (Turner et al., 2003; Pascual et al., 2017).

Various conceptual frameworks e.g. the Millennium Ecosystem Assessment framework (MEA, 2005), the Economics of Ecosystem and Biodiversity (TEEB, 2010) and the Intergovernmental Platform on Biodiversity and Ecosystem Services (IPBES, Díaz et al., 2015) have been developed in recent decades for classifying ecosystem services and linking the impact of changes in supply of ecosystem services caused by combined drivers of change (either natural or human) on human wellbeing, or people’s quality of life.

Ecosystem services frameworks conceptualise the link between humans and nature, through integrating data from multiple sources to establish links between the natural environment and society (Vallecillo et al., 2019). Such frameworks enable the valuation of trade-offs, or of costs and benefits to be included in cost–benefits analysis, to guide and support more informed policy decisions regarding the different management options of multiple ecosystem services, taking into consideration the institutional and cultural context of the ecosystem services beneficiaries (Fisher et al., 2009; Cabral et al., 2015).

**Fig. 3:**
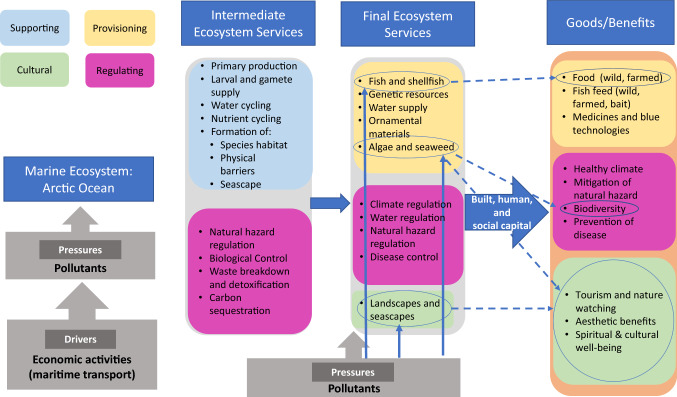
An Ecosystem Services Approach adapted from UK NEA Follow-on (2014) to link the effects of human-induced pressures (pollutants) on Arctic marine and coastal ecosystems services. The blue solid line arrows show the direct impacts of pollutants; the dotted blue arrows represent the indirect impact

In this paper, we adopted the UK National Ecosystem Assessment Follow-on (UK NEA, 2014) conceptual framework to illustrate how human activities (maritime transport as an example) and associated pressures (chemical pollutants) can hamper the capacity of Arctic marine ecosystems to supply services and, ultimately to negatively impact human welfare (Fig. 3). This framework was deemed the most suitable since it addresses specifically marine and coastal systems (Ivarsson et al., 2017).

Environmental contamination caused by maritime transport may have a direct adverse impact on Arctic fish fauna (*fish and shellfish* provisioning final service) and lead to reductions in fish populations (CAFF, 2015; Carroll et al., 2018) as shown by the blue solid arrow in Fig. 3, causing profit losses for the fishing sector (Hasselström et al., 2012; CAFF, 2015). Marine pollution may also have direct impacts (solid blue arrows in Fig. 3) on kelp forests along Arctic coastlines and subsequent indirect impacts (dotted blue arrow) on the provision of multiple ecosystem services that they support (CAFF, 2015). For example, kelp forests provide shelter to many diverse species and are a valuable habitat for a variety of organisms that are, in turn, important as food for fish, birds and mammals, and they also have cultural value for some Arctic communities (Weinke and Amsler, 2012; Christie et al., 2019). Accidental release of pollutants attributable to marine traffic, either for commercial or recreation purposes, may have direct negative impacts on the provision of the final service *landscape and seascape* (as shown by the solid blue arrow in Fig. 3) that benefits Arctic residents as well as tourists worldwide (CAFF, 2015) and subsequently indirectly affect the delivery of cultural benefits (e.g. nature watching) linked to Arctic ecosystems (dotted blue arrow in Fig. 3). Results of a study carried out by Kalternborn (1998) indicate that residents of the islands of Svalbard (Norwegian Arctic) would not remain indifferent to oil spills along the coast, and would also lead to considerable disruption for the tourism industry. Contamination of coastal areas can, therefore, lead to substantial negative socio-economic impacts on the tourism and recreational industries (Hasselström et al., 2012).

This example of the effects of maritime traffic illustrates the complexity of how one pressure can affect the provision of multiple services and associated benefits to society, and demonstrates the importance of using approaches and tools in which ecological, economic and social systems are linked to support decision making (e.g. see Hooper et al., 2017; Ivarsson et al., 2017; Culhane et al., 2019).

## Discussion

Significant efforts have been made in existing Arctic monitoring programmes (e.g. AMAP) and research initiatives (e.g. NERC CAO, Coastal Pollution Toolbox) to describe, explain and predict environmental changes due to different pollution pressures for the Arctic ecosystem. However, the current prediction capacity of available in silico tools is mostly limited by the quality and quantity and of physicochemical property data for pollutants, a limited knowledge of their toxic, including sub-lethal, effects on a wide range of species and across generations, and a limited understanding of exposure across food webs (Nilsen et al., 2019). Moreover, practically no available models allow multiple pollution pressures that span nutrient and organic/inorganic contaminants to be investigated, or the resulting information to be used to inform sustainable environmental management decisions.

Under multiple pressures and climate change conditions, there are complex interactions which need to be teased out. These include changing diets, distributions and behaviour of an array of marine organisms and the knock-on effects on ecosystem services. A combination of observations, experiments, EwE and ecosystem services assessments can integrate potential ecosystem effects from multiple pressures including pollutants and bring us towards an understanding of the impacts associated with multiple pressures in the Arctic. As such, we suggest that EwE/Ecotracer can serve as a screening-level risk characterisation tool to bring together data on hazard, exposure and effects, and assess the relative impacts of a pollutant and other pressures acting on an ecosystem for different species, under dynamic environmental conditions. A particular strength to EwE/Ecotracer over other chemical exposure and impact models is that spatiotemporal dynamics of trophic interactions within the marine ecosystem (e.g. coastal, pelagic, benthic) can be simulated to bridge the gap between food web studies and migration/productivity changes that are, for example, caused by changing nutrient inputs and contaminant exposure and effects. This is a key attribute to EwE/Ecotracer, as climate-induced effects on biomass or productivity in lower trophic levels can be simulated over a time-series. This, in turn, may result in a cascade effect on higher trophic level functioning and the consequences to contaminant bioaccumulation patterns and concentrations within biota can be readily simulated.

As with all models, there are limitations to EwE/Ecotracer. These include (i) not accounting for the impact of complex hydrodynamics on chemical fate and transport, (ii) the lack of a sediment compartment, (iii) difficulties in simulating complex and highly variable emission patterns on chemical fate and transport, (iv) the role of microbes in nutrient/energy cycling and (v) not simulating more than one chemical per model run. External limitations that affect the utility of EwE/Ecotracer include lack of knowledge of sub-lethal effects of many chemicals and how these might affect reproduction, growth, etc. Moreover, EwE/Ecotracer has high data requirements and invokes assumptions about the physicochemical properties of pollutants and process kinetics, which are typically gathered from field campaigns, experiments and/or other models (Christensen and Walters, 2004, 2005). For emerging pollutants, this highlights the need for experimental data to back up and validate models, e.g. by using controlled laboratory experiments to determine the necessary physical–chemical property data (Garnett et al., 2019; Thomas et al., 2021), and especially specific pressure–receptor–effect relationships for Arctic ecosystems. To this end, EwE/Ecotracer could be coupled to (i) quantitative Adverse Outcome Pathway models that elucidate key toxicity mechanisms, as well as (ii) to eco-epidemiological studies based on monitoring data, using the experimental capabilities of the omics approach.

Some of the key questions that we consider still need answering by analysis of multiple pressures are as follows:

- The impacts of accumulation of metals and organic chemicals on vulnerable and protected species and commercial fish, and how these affect protected species, food webs, tourism and human health;
- The impacts of increased terrestrial nutrient inputs on marine ecosystem productivity and greenhouse gas emissions; and
- How changes to migratory behaviour of marine species affect exposure to organic pollutants, in response to local productivity changes, and the subsequent impacts on human health.

Other research priorities which could further our knowledge of the impacts of pollutants and other multiple pressures are as follows:

- Monitoring and assessing the current releases of chemicals, notably from within Arctic sources;
- Emerging pollutants—the properties of these chemicals may not be covered by existing hazard criteria/regulations;
- The effects of changing temperature, seawater pH and the cryosphere on chemical behaviour and their likely toxicity;
- Changes in algal bloom timing in combination with increased pollutant and nutrient loads and associated ecosystem productivity;
- Vulnerable communities and ecosystems that are at risk of impacts from changes in the Arctic ecosystem; and
- Documenting ‘goods and services’ provided to humans by a healthy Arctic marine environment—with direct links to other sectors (e.g. fisheries, tourism, etc.).

In summary, further work is vital to understand the interplay of multiple stressors in the Arctic and advance our understanding of their influences on marine species and ecosystems. Based on an example of pollutant exposure from maritime traffic in the Arctic, we can see that it is important to also consider the scale of these changes from local, to regional and to an ecosystem scale. There are already efforts underway to assess the effects of contaminants on fish and Arctic wildlife, and previous work has emphasised the need to assess the legacy of chemicals for Arctic biota (AMAP, 2016). However, complementary efforts are needed to understand the many interactions, responses and processes at play.

## Societal and policy implications

The wellbeing of present and future generations depends on the state of the natural environment they will inherit. Policy makers in the Arctic thus need to sensibly manage the trade-offs between economic activities and conservation of key marine habitats. Assessment of the multiple services that ecosystems provide is required to support decision making and, ultimately, improve the environment and human welfare (Turner et al., 2003; Guerry et al., 2015).

We propose three steps to answering policy and management questions relating to impacts of multiple pressures including pollutants in the Arctic:

1. Use ecosystem models to screen the observed environmental pressures and interactions of the relevant determinants of ecosystem responses.
2. Explore potential socio-economic impacts of these ecosystem responses for individual ecosystems and ecosystem services.
3. Produce targeted policy relevant documents to support the integration of societal needs. This includes information on the pressures, interactions, sensitivities and linked ecosystem services to inform an interactive decision-making regulatory framework, in support of policy and management.

Further efforts should focus on balancing the ecological, economic and societal interests of the Arctic (Jouffray et al., 2019). There should be a push to harmonise the Arctic initiatives to ensure complementarity of specific policy measures and dedicated frameworks (AMAP, 2016). The methods suggested here can be used to direct current efforts to support the advancement of

- Testing hypothesis-driven questions, such as experimental ice chamber studies;
- Integrated modelling approaches which include feedback effects;
- A centralised data repository for the region;
- Standardised experimental reporting;
- The use of different scales to assess ecological responses;
- Identification of ecosystem processes and services;
- Development of relevant policy frameworks; and
- Integrated funding streams (e.g. in support of ecosystem-level campaigns).

The proposed actions and tools discussed in this paper are applicable to many regions of the world which are under stress from human activities. However, more than anywhere else in the world, access to, and pressures on, the Arctic are increasing. By using the integrated approach proposed here, we will be closer to being better able to manage risks and reduce impacts to Arctic socio-ecological systems and promote sustainable use and conservation of Arctic resources.
